# Streamlining mouse genome editing by integrating AAV repair template delivery and CRISPR-Cas electroporation

**DOI:** 10.1038/s41684-024-01363-w

**Published:** 2024-04-10

**Authors:** Natalia Moncaut

**Affiliations:** Genome Editing and Mouse Models, Cancer Research UK Manchester Institute, The University of Manchester, Manchester, UK

**Keywords:** Biological techniques, Genetics

## Abstract

Recent genome editing techniques have substantially simplified the generation of genetically modified mice. A new study combines adeno-associated viruses (AAV) and electroporation to generate a robust pipeline to deliver CRISPR-Cas reagents into mouse embryos.

Genetically modified mice are indispensable tools for investigating gene function, modelling development and understanding normal physiology and disease. Since 2013, the use of the CRISPR-Cas system as a genome editing technology has sparked a profound transformation in the field of mouse transgenesis^1,2^. Genetic modification has become relatively efficient through the utilization of Cas9 protein (or Cas9 mRNA), single guide RNA (sgRNAs) and various types of repair templates such as single-stranded DNA (ssDNA), linearized or circular double-stranded DNA and long ssDNA (lssDNAs). These modifications enable site-specific insertion of small protein tags, point mutations, fluorescent reporters and recombinases^2^.

Despite the high efficiencies of the CRISPR-Cas systems, editing the mouse genome still utilises the technically demanding, labour-intensive, time-consuming and low-throughput methods of zygote microinjection or embryonic stem (ES) cell targeting. However, in 2015, zygote electroporation (EP) emerged as a highly efficient method for delivering CRISPR-Cas reagents into embryos^3,4^. Since then, many labs have adopted this effective, technically simple and high-throughput strategy. Various types of electroporators can be utilised, enabling the EP of up to 100 embryos with intact zona pellucida in a single round, with a high survival rate attributed to less physical damage compared to microinjection. However, larger genome modifications which rely on the use of long double-stranded DNAs or lssDNAs often result in lower knock-in efficiencies. This limitation is likely due to EP being inefficient in delivering large repair templates into zygotes.

It has been shown that mouse embryos can also be transduced by adeno-associated viruses (AAV) carrying Cas9 and sgRNAs^5,6^. Building on this, Chen et al.^7^ employed the full cargo capacity of AAV (4.7kb) to deliver large repair templates for complex mouse genome engineering, followed by the EP of Cas9 protein and sgRNAs complex (referred to as AAV-EP). This combined approach has been extensively and efficiently applied at Ben Davies’ Genetic Modification Service at The Francis Crick Institute, as described in a recent publication^8^. This new paper presents a detailed workflow for targeting a diverse set of genetic alterations to the mouse genome, including site-specific integration of recombinases, fluorescent proteins, conditional expression cassettes and floxed alleles (Fig. 1). Comparing their method to contemporary microinjection methods used in similar projects, the authors demonstrate a high rate of embryo survival following AAV-EP compared to microinjection alone. This finding suggests that the combination of viral treatment with EP has minimal toxicity and causes less mechanical damage to the embryos.

**Fig. 1:**
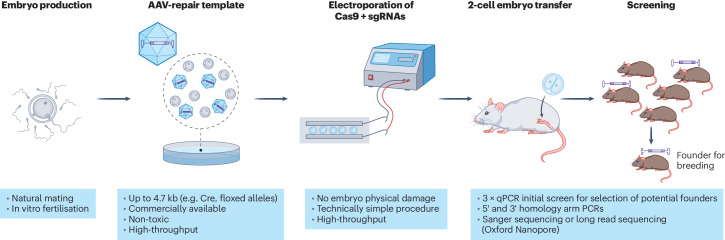
AAV-EP: A robust pipeline to efficiently generate genetically modified mice. Starting with the production of zygotes by natural mating (or alternatively, in vitro fertilisation), the pipeline continues by introducing AAV-repair templates into embryo incubation drops. Next, CRISPR-Cas reagents (including Cas9 protein and sgRNAs) are electroporated into batches of up to 100 embryos, depending on the slide or cuvette size, followed by overnight culture until reaching the 2-cell stage. The surviving embryos are then surgically implanted into foster mice, and the resulting litters are screened for accurate targeting. Potential founders are identified and further bred to establish the new mouse colony.

Additionally, the authors proposed a straightforward yet critical screening method to assess the targeting efficiency of potential founders. They designed three distinct qPCR assays: one for copy number variation (CNV), aiming to detect the targeted allele; another for loss-of-allele (LOA), where the wild-type allele detection should be reduced or undetected if modification was homozygous; and a third assay to detect AAV-backbone integration. This initial qPCR assessment enables the rapid identification and elimination of F0 animals with incorrect genetic modifications, thereby restricting further analysis to a smaller cohort of mice. By using these qPCR assays, the possibility of ectopic integration of AAV and their concatemer recombination can be reliably ruled out. However, a comprehensive validation process still necessitates the verification of newly generated alleles at the sequence level. For this purpose, the authors used positive PCR amplification across the 5’ and 3’ homology arms, followed by Sanger sequencing. While the authors confirmed the absence of mutations in founder mice analysed in this way, it is important to note that AAV production can introduce unwanted point mutations, as observed in lssDNA synthesis^9^. Moreover CRISPR-Cas system, though effective, might introduce incorrect alterations alongside the intended targeting^9,10^. Recently, to overcome the limited length of Sanger sequencing reads (500–800bp), Oxford Nanopore technologies have been used to provide long-read sequencing covering the entire length of the targeting region from a single DNA molecule^11^. It is important to highlight that correct targeting efficiencies exhibit notable variation. The primary source of variation often lies in the complexity of the insertions, with modifications containing endogenous sequences (such as floxed or conditional knock-in alleles) typically displaying lower targeting efficiency.

From the perspective of the 3Rs (Replacement, Reduction, Refinement), Davies’ group compared the cost, in terms of the number of animals required, to generate newly genetically modified mice by using AAV-EP in zygotes versus traditional ES-cell targeting leading to chimera production. This comparison was made across different projects with similar size and complexity of genetic alterations (with AAV packaging size limited to 4.7kb). Interestingly, the new targeting approach significantly reduced the total number of animals needed. The quantity of female donors necessary for blastocyst isolation was more than double the number needed for zygotes, likely because of the limited yield of high-quality blastocysts suitable for ES cell microinjection. In addition, a greater quantity of ES cell-chimera breeding pairs was required compared to the number of founder breeding pairs needed in the AAV-EP zygote route. The AAV-EP method not only demonstrated a substantial reduction in animal use but also in the cost and time required to edit the mouse genome.

Any laboratory or facility involved in producing genetically engineered mice should seriously consider integrating this AAV-EP approach and initial F0 screening methodology. This approach provides an efficient route to target large and complex genetic modifications. Replacing the time-consuming, expensive and technically demanding microinjection and ES-cell targeting approaches with this simpler, more efficient and potentially high-throughput method will undoubtedly decrease the turnaround time for generating new mouse models. This transition streamlines the process, reduces the number of animals required and holds promise for accelerating research and advancing discoveries in many fields.
